# Rumination and psychological resilience in Chinese civil aviation flight students: the chain mediating role of proactive coping and generalized anxiety disorder

**DOI:** 10.3389/fpsyg.2025.1606045

**Published:** 2025-08-01

**Authors:** Mingyu Liao, Haozhe Wang, Zongyu Liu, Enliang Hu

**Affiliations:** ^1^Department of Physical Training, Institute of Aviation Safety and Security, China Civil Aviation Flight Academy, Chengdu, China; ^2^School of Physical Education, China University of Mining and Technology, Jiangsu, China; ^3^Department of Sport and Exercise Science, College of Education, Zhejiang University, Hangzhou, China

**Keywords:** rumination, psychological resilience, generalized anxiety disorder, proactive coping, chain mediating effect

## Abstract

**Background:**

As the focus of aviation safety shifts from technology to human factors, the central role of psychological resilience in flight safety has become increasingly prominent. However, the internal mechanism of how rumination affects psychological resilience, particularly the chain mediating effect of proactive coping and generalized anxiety, has not been thoroughly explored in high-stress populations.

**Methods:**

Employing a cross-sectional design, 1,235 flight students from the Civil Aviation Flight University of China were surveyed using the Ruminative Response Scale (RRS), Connor-Davidson Resilience Scale (CD-RISC), Simplified Coping Style Questionnaire (SCSQ), and Generalized Anxiety Disorder Scale (GAD-7) to gather data. Structural equation modeling (SEM) and the Bootstrap method were used to test the chain mediating effect.

**Results:**

Rumination exhibited a significant negative correlation with psychological resilience (*r* = −0.365, *p* < 0.01) and proactive coping (*r* = −0.285, *p* < 0.01), and a positive correlation with generalized anxiety (*r* = 0.337, *p* < 0.01). Psychological resilience showed a positive correlation with proactive coping (*r* = 0.727, *p* < 0.01) and a negative correlation with generalized anxiety (*r* = −0.270, *p* < 0.01). Mediation effect analysis revealed that proactive coping and generalized anxiety each played a partial mediating role between rumination and psychological resilience. The mediating effect comprised three paths: “rumination → proactive coping → psychological resilience,” “rumination → generalized anxiety → psychological resilience,” and “rumination → proactive coping → generalized anxiety → psychological resilience,” with effect sizes of 91.22, 7.80, and 0.98%, respectively.

**Conclusion:**

Rumination in civil aviation flight students not only directly impairs psychological resilience but also indirectly influences it through the chain mechanism of inhibiting proactive coping and intensifying generalized anxiety. Consequently, it is recommended that civil aviation psychological training focus on cognitive-behavioral interventions to interrupt the activation of rumination, foster adaptive coping strategies, and construct a psychological resilience development model tailored to the specific demands of the aviation profession.

## 1 Introduction

The evolution of the aviation safety system represents the historical interplay between human cognitive capabilities and the risks inherent in complex systems. With the continuous enhancement of aircraft airworthiness standards, global aviation accidents have decreased since the 1960s (Kelly and Efthymiou, 2019). However, safety hazards stemming predominantly from human factors have presented new contemporary challenges (de Sant and de Hilal, 2021). Accident analyses indicate that human factors contribute to approximately 70% of aviation accidents, wherein psychological factors are a primary component (Li et al., 2002). According to data from the Civil Aviation Administration of China (CAAC), neuropsychiatric issues rank as the second leading cause for the medical disqualification of flight students, accounting for 20.1% of cases (Yongtang and Ce, 2024). These statistics underscore the central role of psychological resources in aviation safety.

Psychological resilience refers to the process of positive adaptation in the face of adversity, trauma, or significant life stress, signifying an individual’s capacity to “bounce back” from life’s pressures and setbacks (Windle, 2011). Research on psychological resilience has largely concentrated on populations such as children and adolescents (Zhao et al., 2023), military personnel (Sefidan et al., 2021), and healthcare workers (Devi et al., 2021; Walpita and Arambepola, 2020). Psychological resilience is not only a core psychological resource for coping with stress but also constitutes a critical element of a pilot’s psychological constitution.

Currently, research on psychological resilience among pilots is limited. For instance, Chen and Han (2018), through theoretical analysis, developed and tested a hypothetical model on the influence of psychological resilience on pilots’ safety behaviors using structural equation modeling. Their findings revealed a significant negative correlation, where higher levels of psychological resilience were associated with fewer unsafe behaviors. Furthermore, a 10-week longitudinal study on general aviation pilots by Zhang et al. (2017) demonstrated that improvements in psychological resilience and reductions in psychological stress significantly enhanced pilots’ safety performance. Therefore, investigating the mechanisms that influence psychological resilience in civil aviation flight students and identifying effective interventions to cultivate it are of direct consequence to their flight training efficacy and future aviation safety.

### 1.1 The relationship between rumination and psychological resilience in civil aviation flight students

The Conservation of Resources (COR) theory posits that individuals cope with stress by continuously managing their balance of psychological resources; once resource loss occurs, it becomes difficult to reverse, representing a key factor in the decline of psychological resilience (Hobfoll, 1989). Rumination, a pattern of persistent and repetitive negative thought (Nolen-Hoeksema, 1987), depletes executive function resources (Whitmer and Gotlib, 2013) and reinforces threat-perception biases (Mclaughlin and Nolen-Hoeksema, 2011), thereby trapping individuals in a vicious cycle of cognitive and emotional resource depletion.

Within specific occupational groups, these cognitive patterns can be exacerbated by the unique nature of environmental stressors. For instance, studies on air force personnel (Zhao et al., 2020) and emergency medical staff (Gillespie and Gates, 2013) indicate that long-term exposure to high-intensity stressors reshapes an individual’s cognitive-emotional regulation patterns. To date, research on the effects of rumination has primarily focused on populations such as university students, individuals diagnosed with or recovering from depression, and those with other mental disorders, revealing distinct characteristics of rumination across these groups (du Pont et al., 2019; Qiu et al., 2023; Satyshur et al., 2018).

In high-stress populations, however, unique stress exposure and occupational characteristics may alter the functional nature of rumination. For example, research on healthcare workers in makeshift field hospitals during the COVID-19 pandemic revealed that psychological resilience plays a critical mediating role between rumination and post-traumatic growth (PTG) (Liu et al., 2023; Yi et al., 2024). Similarly, among firefighters, rumination has been shown to exacerbate psychological exhaustion and burnout (Yang and Ha, 2019). Nevertheless, these studies have largely investigated single mediating pathways, such as resilience or anxiety, with samples concentrated in medical or emergency response fields. Consequently, the dynamic pathways through which rumination operates within the distinct human factors context of civil aviation remain largely unexplored.

### 1.2 The potential mediating role of proactive coping between rumination and psychological resilience in civil aviation flight students

Coping styles are defined as the cognitive and behavioral efforts individuals employ to manage specific internal or external demands that are appraised as taxing or exceeding their resources (Ray et al., 1982). As a future-oriented approach, proactive coping is conceptualized as the effort to build general resources that facilitate the pursuit of challenging goals and promote personal growth (Schwarzer and Taubert, 2002). Research has identified proactive coping as a significant protective factor for psychological resilience among firefighters (Wagner and Martin, 2012). Similarly, a study on civil aviation pilots by Xu et al. (2022a) found that proactive coping not only directly and positively predicted mental health but also exerted an indirect protective effect by reducing perceived stress (Meng et al., 2011; Yang et al., 2013). This provides empirical support for the mechanisms of coping strategies in high-stress contexts.

Coping is considered a core regulatory variable in the stress process (Folkman et al., 1986; Folkman and Moskowitz, 2004), and coping strategies are regarded as antecedents of resilience (Rutter, 2007). The role of coping has received substantial empirical support in high-stress populations. According to the cognitive-transactional model of stress and coping (Lazarus and Folkman, 1984), rumination can impair the effectiveness of problem-focused coping, leading individuals into a state of passive reactivity to stress. The chronic stressors inherent in the flight training environment—such as its enclosed nature, high cognitive workload, and stringent evaluation standards—may intensify flight students’ tendency to ruminate on negative events. This ruminative cognitive pattern is hypothesized to not only directly deplete psychological resources but also to weaken psychological resilience by inhibiting the use of proactive coping strategies. Notably, while the direct relationship between proactive coping and psychological resilience is well-documented, the potential mediating role of proactive coping in the relationship between cognitive patterns like rumination and resilience remains insufficiently explored.

### 1.3 The potential mediating role of generalized anxiety between rumination and psychological resilience in civil aviation flight students

Generalized Anxiety Disorder (GAD) is a mental health condition characterized by persistent, excessive, and pervasive anxiety and worry that is not attached to any specific object or situation (Showraki et al., 2020). It is often accompanied by somatic and autonomic nervous system symptoms (Citkowska-Kisielewska et al., 2019; First, 2013). Generalized anxiety is particularly prominent among adolescent populations, tends to be exacerbated by stress, and can be complicated by numerous factors (First, 2013; Kertz and Woodruff-Borden, 2011; Kim and Shin, 2022; Liu, 2020; Logue et al., 1993; Romera et al., 2010). According to the cognitive model of anxiety (Beck and Clark, 1997), rumination induces a persistent state of anxious arousal by amplifying catastrophic interpretations of ambiguous threats. Furthermore, studies have established a significant correlation between generalized anxiety and psychological resilience (Pechmann et al., 2014; Sheerin et al., 2018).

### 1.4 The potential chain mediating effect of proactive coping and anxiety between rumination and psychological resilience

Rumination is a maladaptive emotion regulation strategy characterized by a persistent focus on negative thoughts and repetitive contemplation of their causes and consequences without progressing toward active problem-solving (Eldeleklioğlu, 2015; Stone et al., 2016). As a process reflecting negative emotional and cognitive states (Barrocas et al., 2015; Selby et al., 2010), rumination has been linked in multiple studies to adverse outcomes, including heightened emotional dysregulation, impulsivity, substance abuse, perfectionistic tendencies (Mansueto et al., 2022; Mansueto et al., 2024), eating psychopathology (Palmieri et al., 2021; Palmieri et al., 2023), increased shyness (Palmieri et al., 2018), and depressive symptoms (Nolen-Hoeksema, 2000).

The Conservation of Resources (COR) theory posits that individuals strive to acquire, protect, and maintain psychological resources to manage stress, and that a “loss spiral” can precipitate a significant decline in adaptive functioning. Within this framework, proactive coping is considered a key psychological resource (Halbesleben et al., 2014). We hypothesize that the depletion of this resource may, through a subsequent escalation in anxiety, initiate a cascading decline in psychological resilience.

Previous research provides support for the links in this proposed chain. Proactive coping has been shown to reduce levels of anxious arousal via cognitive restructuring (Goldfried and Trier, 1974) and problem-focused strategies (Seligman et al., 2005). Conversely, the accumulation of anxiety can impair the ability to mobilize proactive coping resources (Pang et al., 2021), creating a vicious cycle of “resource investment depletion” (Hobfoll, 2001). Furthermore, research has consistently demonstrated a stable negative association between levels of anxiety and psychological resilience (Benetti and Kambouropoulos, 2006; Shi et al., 2016).

### 1.5 Aims of the present studies

This study employs the Conservation of Resources (COR) Theory as its core theoretical framework to explore the depletion mechanism of psychological resilience as a critical resource among civil aviation flight students undergoing high-demand training. The COR framework posits that rumination is a core driver of “resource loss spirals.” We propose that rumination not only directly consumes an individual’s cognitive and emotional resources but also systematically erodes psychological resilience through a cascading mechanism. Specifically, an initial loss of resources is thought to inhibit proactive coping, which in turn amplifies threat perception, thereby fostering and exacerbating generalized anxiety and accelerating further resource depletion.

Furthermore, testing this model within a cohort of Chinese civil aviation flight students holds particular theoretical and practical significance. The cultural concept of “face” (mianzi) may intensify the sense of resource loss following failure, potentially amplifying the intensity and detrimental effects of rumination. To isolate the relationships between our core constructs, we included age, academic year, family residence (urban vs. rural), only-child status, and smoking and drinking habits as control variables.

Based on the foregoing, we propose the following hypotheses:

*H1*: Rumination will be negatively associated with psychological resilience in civil aviation flight students.

*H2*: Proactive coping will mediate the relationship between rumination and psychological resilience.

*H3*: Generalized anxiety will mediate the relationship between rumination and psychological resilience.

*H4*: Proactive coping and generalized anxiety will have a chain mediating effect on the relationship between rumination and psychological resilience. Specifically, rumination will be associated with lower proactive coping, which in turn will be associated with higher generalized anxiety, ultimately leading to lower psychological resilience.

## 2 Materials and methods

### 2.1 Participants and procedure

This cross-sectional study collected data via a questionnaire survey administered to civil aviation flight students at the Civil Aviation Flight University of China (CAFUC). Given the students’ training and administrative structure, a two-stage cluster sampling method was employed, with academic classes serving as the clusters. The procedure was as follows: First, a comprehensive list of all first- and second-year classes was obtained. Second, using simple random sampling, a subset of classes was selected from within each academic year (stratum). Third, all students within the selected classes were invited to participate in the survey.

As of the survey date (December 24, 2024), the total enrolled population consisted of 2,613 students: 1,487 first-year (56.9%) and 1,126 s-year (43.1%). The final sample comprised 1,235 students, including 727 first-year (58.9%) and 508 s-year (41.1%) students. A Chi-square goodness-of-fit test confirmed that there was no significant difference between the sample and population distributions by academic year [χ^2^(1) = 1.95, *p* = 0.163], indicating the sample is representative of the student population in this regard.

Data were collected using an online platform. Inclusion criteria were: (1) being a currently enrolled student at CAFUC and (2) providing voluntary consent to participate. Initially, 1,296 questionnaires were completed. Following data screening, responses were excluded for incompleteness, exceptionally short completion times (<200 s), or patterned responses (e.g., selecting the same option for ≥10 consecutive items). This resulted in a final valid sample of 1,235 (a valid response rate of 95.29%). The survey instrument included measures of rumination, psychological resilience, proactive coping, and generalized anxiety.

This study was approved by the Ethics Committee of the Civil Aviation Flight University of China (Approval No. 20240014). The research team received standardized training to ensure consistency in data collection. Prior to participation, all individuals were informed of the study’s purpose, procedures, and their right to withdraw at any time. Informed consent was obtained electronically from all participants before they could access the questionnaire.

### 2.2 Measures

#### 2.2.1 Ruminative Response Scale

The Ruminative Response Scale, developed by Nolen-Hoeksema in 1991 based on the Response Styles Theory, consists of 3 dimensions and 22 items. Items 1, 2, 3, 4, 6, 8, 9, 14, 17, 18, 19, and 22 belong to the symptom-focused rumination factor; items 5, 10, 13, 15, and 16 belong to the brooding factor; and items 7, 11, 12, 20, and 21 belong to the reflection factor. This study used the Chinese version translated by Han and Yang (2009). The scale employs a 4-point Likert scale, with scores ranging from 1 to 4, corresponding to “never,” “sometimes,” “often,” and “always.” Higher scores indicate a more severe tendency toward rumination. In this study, the Cronbach’s *α* coefficient for the scale and its dimensions was 0.951.

#### 2.2.2 Connor-Davidson Resilience Scale

Psychological resilience was measured using the Chinese version of the Connor-Davidson Resilience Scale, originally developed by Connor and Davidson in 2003 and translated and revised by Yu and Zhang (2007). This scale is primarily used to describe and measure the positive abilities that promote an individual’s adaptation to adversity. The scale consists of 25 items, including three dimensions: tenacity (13 items), strength (8 items), and optimism (4 items). A 5-point Likert scale is used, with scores ranging from 0 to 4, representing “not true at all,” “rarely true,” “sometimes true,” “often true,” and “true nearly all the time.” The total score ranges from 0 to 100, with scores <45 indicating a low level of psychological resilience, 45–65 indicating a moderate level, and >65 indicating a high level. In this study, the Cronbach’s *α* coefficient for the scale and its dimensions was 0.975.

#### 2.2.3 Simplified Coping Style Questionnaire

In recent years, several coping measures have been developed for the Proactive Coping Questionnaire. Among them, the 66-item Ways of Coping Questionnaire (Folkman and Lazarus, 1985) and the 30-item Coping Strategies Questionnaire (Carver et al., 1989) are the most widely used versions. However, Chinese researchers found that these versions were not suitable for the Chinese population due to inconsistent factor analysis results. Therefore, we used the Simplified Coping Style Questionnaire (SCQ) (Xie, 1998) in this study. The SCQ contains 20 items that measure two coping styles: positive coping, which assesses the strategies or methods employed by the respondent when dealing with emotions or problems. The scale includes two dimensions, positive and negative, with 12 and 8 items, respectively, for a total of 20 items. Each item is scored as “0 points,” “1 point,” “2 points,” and “3 points” for “never used,” “occasionally used,” “sometimes used,” and “frequently used,” respectively. The total score for positive coping is the sum of scores from item 1 to item 12, with higher scores indicating a greater tendency to adopt positive coping styles (Yu et al., 2014). In this study, the Cronbach’s *α* coefficient for the positive coping dimension was 0.944.

#### 2.2.4 Generalized Anxiety Disorder Scale-7

This scale requires respondents to recall the frequency of anxiety-related symptoms over the past 2 weeks (Sun et al., 2021). It contains 7 items, each rated on a scale of 0–3, corresponding to “not at all,” “several days,” “more than half the days,” and “nearly every day.” The total score ranges from 0 to 21, with higher scores indicating more severe anxiety symptoms. In this study, the Cronbach’s α coefficient for the scale was 0.954.

### 2.3 Statistical analysis

Data were processed using SPSS 26.0 (IBM Corporation, Armonk, New York) and AMOS 28.0 (IBM Corporation, Armonk, New York), and the validity of the scales was confirmed before use. Descriptive statistics included calculating means and standard deviations, and Pearson correlation analysis was used to reveal the correlations between variables. Through linear regression, with rumination as the independent variable and psychological resilience, proactive coping, and generalized anxiety disorder as the dependent variables, the predictors of the dependent variables were determined. This study used the SPSS macro Process, developed by Andrew F. Hayes, to test the mediation effects. By selecting Model 6 and analyzing 5,000 sample data points, the 95% confidence interval of the mediation effects was estimated. Additionally, following Hayes’ recommendations, this study controlled for demographic variables such as gender and age as mediating roles in the test. After the SPSS analysis, structural equation modeling (SEM) was performed using AMOS 28.0 to examine the impact of rumination on psychological resilience and the interrelationships between proactive coping and generalized anxiety disorder among civil aviation flight students.

## 3 Results

### 3.1 Common method bias test

The data collection in this study was conducted through online self-assessment questionnaires on Questionnaire Star, which may introduce common method bias. Harman’s single-factor test was used to perform factor analysis on all items involved in this study. According to Harman’s single-factor test, exploratory factor analysis extracted a total of 7 factors with eigenvalues greater than 1, and the first factor explained 25.806% of the variance, which is lower than the critical value of 40%, indicating that the impact of common method bias in this study was not significant.

### 3.2 Demographic characteristics

Table 1 summarizes the sample characteristics. The age of the 1,235 civil aviation flight students was 18.81 ± 0.79 years, ranging from 17 to 24 years old. Among these participants, in terms of grade level, there were 727 freshmen (58.9%) and 508 sophomores (41.1%). Regarding family residence, 1,048 participants (84.9%) were from urban areas, and 187 (15.1%) were from rural areas. In terms of being an only child, 670 participants (54.3%) were only children, while 565 (45.7%) were not. Regarding smoking or drinking habits, 10 participants (0.8%) only smoked, 32 (2.6%) only drank alcohol, 33 (2.7%) had both habits, and 1,160 (93.9%) had neither habit.

**Table 1:** Socio-demographic characteristics of the participants.

Variables	Sociodemographic characteristics	Participants (*N* = 1,235)
Age, Mean (SD)		18.81 ± 0.790
Grade N(%)	Freshman	727(58.9%)
	Sophomore	508(41.1%)
Urban–rural provenance, N(%)	Urban	1,048(84.9%)
	Rural	187(15.1%)
Only child N(%)	Yes	670(54.3%)
	No	565(45.7%)
Smoking or drinking N(%)	Smoking only	10(0.8%)
	Drinking only	32(2.6%)
	Both	33(2.7%)
	Neither	1,160(93.9%)

### 3.3 Descriptive statistics

This study conducted descriptive statistical analysis on variables such as rumination and its dimensions, psychological resilience and its dimensions, generalized anxiety disorder, and proactive coping. The results are shown in Table 2. The total score for rumination (Ru) was 31.68 ± 9.919. Among its subscales, symptom rumination had the highest score (15.75 ± 5.046), followed by compulsive thinking (8.08 ± 2.855) and self-reflection and deep contemplation (7.85 ± 2.923), indicating that the main characteristic of rumination in civil aviation flight students is symptom rumination. The value of psychological resilience (PR) was 80.25 ± 15.194, which, according to the scale standards, is at a high level (65 points), indicating that the research subjects generally have good psychological resilience. Specifically, resilience (Re) was 42.12 ± 7.930; self-reliance (Sr) was 28.30 ± 5.682; and optimism (Op) was 9.82 ± 2.120. This indicates that among the three dimensions of psychological resilience, the scores for resilience and self-reliance are relatively high, while the score for optimism is relatively low. The value of generalized anxiety disorder (GAD) was 8.65 ± 3.053, which, according to the scale’s scoring standards, falls within the range of mild anxiety symptoms (5–9 points), indicating that the research subjects generally have a certain degree of anxiety symptoms, but the level is mild. The value of proactive coping (PC) was 28.20 ± 6.859, which is at a medium-high level, indicating that when facing stress and difficulties, the research subjects tend to adopt relatively positive coping methods. Overall, the sample of civil aviation flight students in this study exhibited moderate levels of rumination, high levels of psychological resilience, mild anxiety symptoms, and medium-high levels of proactive coping ability. These characteristics may be closely related to the selection criteria, training process, and occupational characteristics of flight students. Flight students need to have excellent psychological qualities and coping abilities to effectively deal with multiple pressures and challenges in flight training and future work.

**Table 2:** Descriptive statistical analysis.

Variable	N	Mean	SD
Rumination (Ru)	1,235	31.68	9.919
Symptom rumination (SR)	1,235	15.75	5.046
Compulsive thinking (CT)	1,235	8.08	2.855
Self-reflection and deep contemplation (SDC)	1,235	7.85	2.923
Psychological resilience (PR)	1,235	80.25	15.194
Resilience (Re)	1,235	42.12	7.930
Self-reliance (Sr)	1,235	28.30	5.682
Optimism (Op)	1,235	9.82	2.120
General anxiety disorder (GAD)	1,235	8.65	3.053
Proactive coping (PC)	1,235	28.20	6.859

### 3.4 Correlation analysis of rumination, psychological resilience, proactive coping, and generalized anxiety disorder in civil aviation flight students

**Figure 1:**
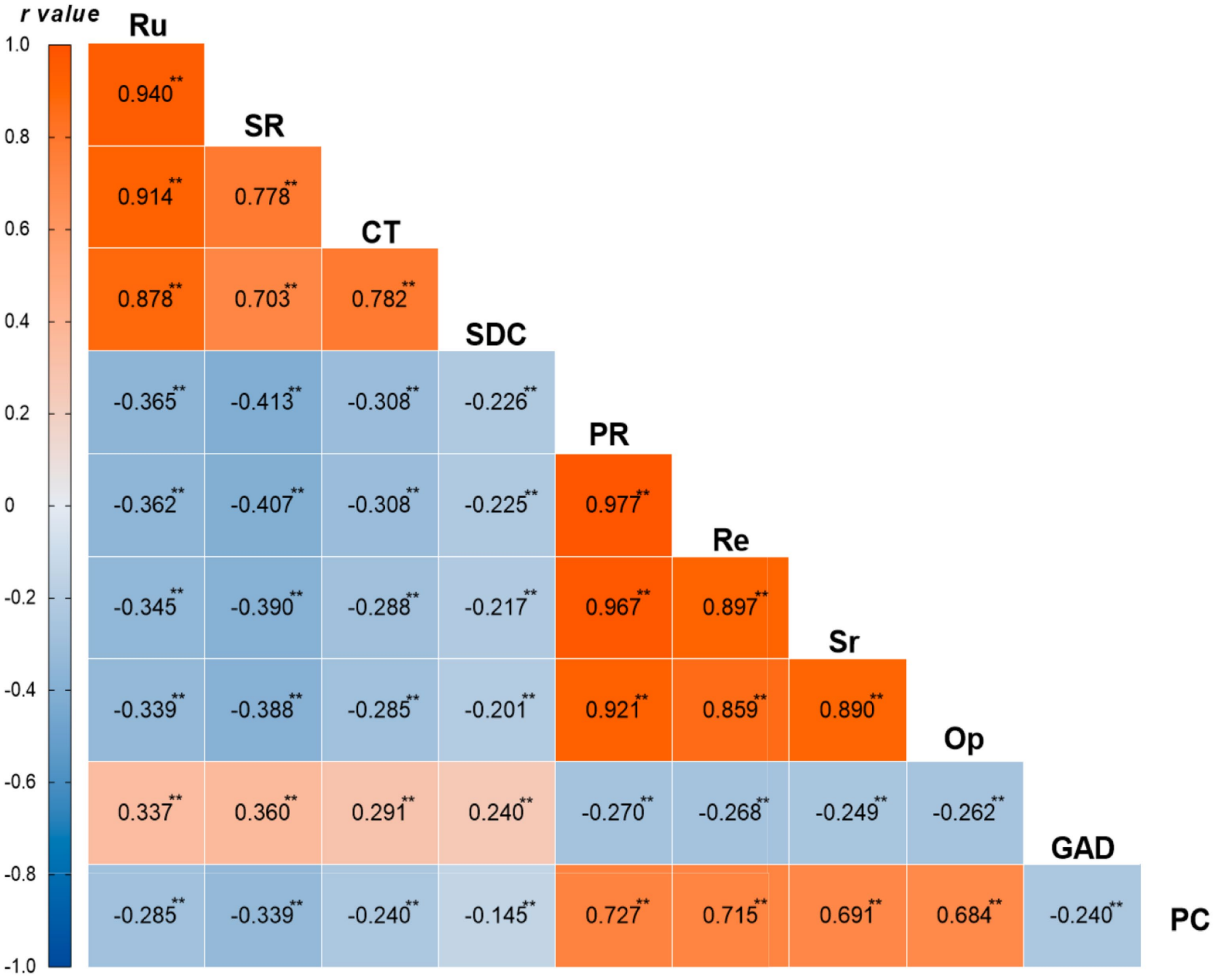
Correlation coefficient heatmap. **p < 0.01. Ru, Rumination; SR, Symptom rumination; CT, Compulsive thinking; SDC, Self-reflection and deep contemplation; PR, Psychological resilience; Re, Resilience; Sr, Self-reliance; Op, Optimism; GAD, Generalized Anxiety Disorder; PC, Proactive Coping.

This study conducted Pearson correlation analysis on each variable (Figure 1). The results showed that there was a high positive correlation between rumination (Ru) and its various dimensions: Symptom rumination, Compulsive thinking, and Self-reflection and deep contemplation (*r* = 0.703–0.940, *p* < 0.01). Rumination was significantly negatively correlated with psychological resilience and its dimensions (*r* = −0.201 to −0.413, *p* < 0.01), significantly positively correlated with generalized anxiety disorder (*r* = 0.240–0.360, *p* < 0.01), and significantly negatively correlated with proactive coping (PC) (*r* = −0.145 to −0.339, *p* < 0.01). Psychological resilience and its various dimensions, Resilience, Self-reliance, and Optimism, were highly positively correlated (*r* = 0.859–0.977, *p* < 0.01), significantly negatively correlated with generalized anxiety disorder (*r* = −0.249 to −0.270, *p* < 0.01), and significantly positively correlated with proactive coping (*r* = 0.684–0.727, *p* < 0.01). Additionally, generalized anxiety disorder was significantly negatively correlated with proactive coping (*r* = −0.240, *p* < 0.01). The correlation analysis indicated that there were significant correlations among the main variables, providing a basis for subsequent chain mediation effect analysis.

### 3.5 Chain mediation effect test

This study first used AMOS 28.0 to perform structural equation modeling to examine the structural relationships among rumination, proactive coping, generalized anxiety disorder, and psychological resilience. The model fit indices were good (GFI = 0.964, AGFI = 0.928, NFI = 0.977, IFI = 0.979, TLI = 0.967, CFI = 0.979, PNFI = 0.628, PCFI = 0.629, RMSEA = 0.086), providing initial support for the path relationships among the variables. This study used Hayes’ PROCESS (version 3.4) in SPSS 26.0 to construct a multiple mediation model, with demographic variables such as age, family residence, and being an only child as control variables. After controlling for demographic variables, the results (Table 3) showed that: (1) rumination had a significant negative predictive effect on proactive coping (*B* = -0.279, *p* < 0.01); (2) rumination had a significant positive predictive effect on generalized anxiety disorder (*B* = 0.289, *p* < 0.01) and a significant negative predictive effect on psychological resilience (*B* = -0.367, *p* < 0.05); (3) after including proactive coping and generalized anxiety disorder, the predictive effect of rumination on psychological resilience weakened but remained significant (*B* = −0.161, *p* < 0.01), psychological resilience had a significant positive predictive effect on proactive coping (*B* = 0.669, *p* < 0.01), and psychological resilience (*B* = −0.056, *p* < 0.01) had a significant negative predictive effect on generalized anxiety disorder.

**Table 3:** Regression analysis of the chain mediation model (*n* = 1,235).

Variables	Proactive coping			Generalized anxiety disorder			Psychological resilience			Psychological resilience		
	*B*	*t*	*β*	*B*	*t*	*β*	*B*	*t*	*β*	*B*	*t*	*β*
Age	0.001	0.021	0.001	−0.013	−0.358	−0.013	0.009	0.243	0.009	0.007	0.290	0.007
Grade	0.067	1.861	0.067	−0.006	−0.183	−0.006	0.023	0.646	0.023	−0.023	−0.922	−0.023
Residence	−0.069*	−2.454	−0.069	0.004	0.145	0.004	−0.088**	−3.197	−0.088	−0.041*	−2.061	−0.041
Only child	−0.025	−0.890	−0.025	−0.009	−0.342	−0.009	0.017	0.633	0.017	0.034	1.723	0.034
Smoking or drinking	0.045	1.634	0.045	−0.052	−1.953	−0.052	0.034	1.265	0.034	0.000	0.023	0.000
Rumination	−0.279**	−10.134	−0.279	0.289**	10.337	0.289	−0.367**	−13.696	−0.367	−0.161**	−7.758	−0.161
Proactive coping				−0.154**	−5.522	−0.154				0.669**	33.249	0.669
Generalized anxiety disorder										−0.056**	−2.770	−0.056
*R* ^2^	0.092			0.139			0.142			0.561		
Adjusted *R*^2^	0.088			0.134			0.138			0.558		
*F*-value	*F* (6, 1,228) = 20.781, *p* = 0.000			*F* (7, 1,227) = 28.328, *p* = 0.000			*F* (6, 1,228) = 33.847, *p* = 0.000			*F* (8, 1,226) = 195.816, *p* = 0.000		

*p < 0.05, **p < 0.01.

The Bootstrap method was further used to test the mediating effects of proactive coping and generalized anxiety disorder between rumination and psychological resilience. As shown in Table 4, the 95% confidence interval was estimated with 5,000 repeated samplings. The results indicated that rumination not only directly affected psychological resilience (direct effect = −0.161, 95% CI [−0.202, −0.121]) but also produced significant effects through three indirect paths. Specifically, the indirect effect of the first path “rumination → proactive coping → psychological resilience” was −0.187 (95% CI [−0.233, −0.141]), accounting for 91.22% of the total indirect effect, *Z* = -7.939, indicating that proactive coping is the main mediating variable for the effect of rumination on psychological resilience. The indirect effect of the second path “rumination → generalized anxiety disorder → psychological resilience” was −0.016 (95% CI [−0.032, −0.003]), accounting for 7.80% of the total indirect effect, *Z* = −2.138, indicating that generalized anxiety disorder also played a significant mediating role between the two variables. The indirect effect of the third path “rumination → proactive coping → generalized anxiety disorder → psychological resilience” was −0.002 (95% CI [−0.006, −0.000]). Although the proportion was small (0.98%), the chain mediating effect still reached statistical significance (*Z* = −1.569). The total indirect effect was −0.205 (95% CI [−0.252, −0.158]), *Z* = −8.550, indicating that the overall mediation effect path was significant.

**Table 4:** Summary of Bootstrap analysis of mediating effects.

Effect types	Item	Effect	*SE*	Boot 95%CI		z	Proportion
				LLCI	ULCI		
Direct effect	Rumination⇒Psychological resilience	−0.161	0.021	−0.202	−0.121	——	—— ——
Indirect effect	Rumination⇒Proactive Coping⇒Psychological resilience	−0.187	0.008	−0.233	−0.141	−7.939	91.22%
	Rumination⇒General Anxiety Disorder⇒Psychological resilience	−0.016	0.008	−0.032	−0.003	−2.138	7.80%
	Rumination⇒Proactive Coping⇒General Anxiety Disorder⇒Psychological resilience	−0.002	0.002	−0.006	−0.000	−1.569	0.98%
Total indirect effect		−0.205	0.024	−0.252	−0.158	−8.550	100%

**Figure 2:**
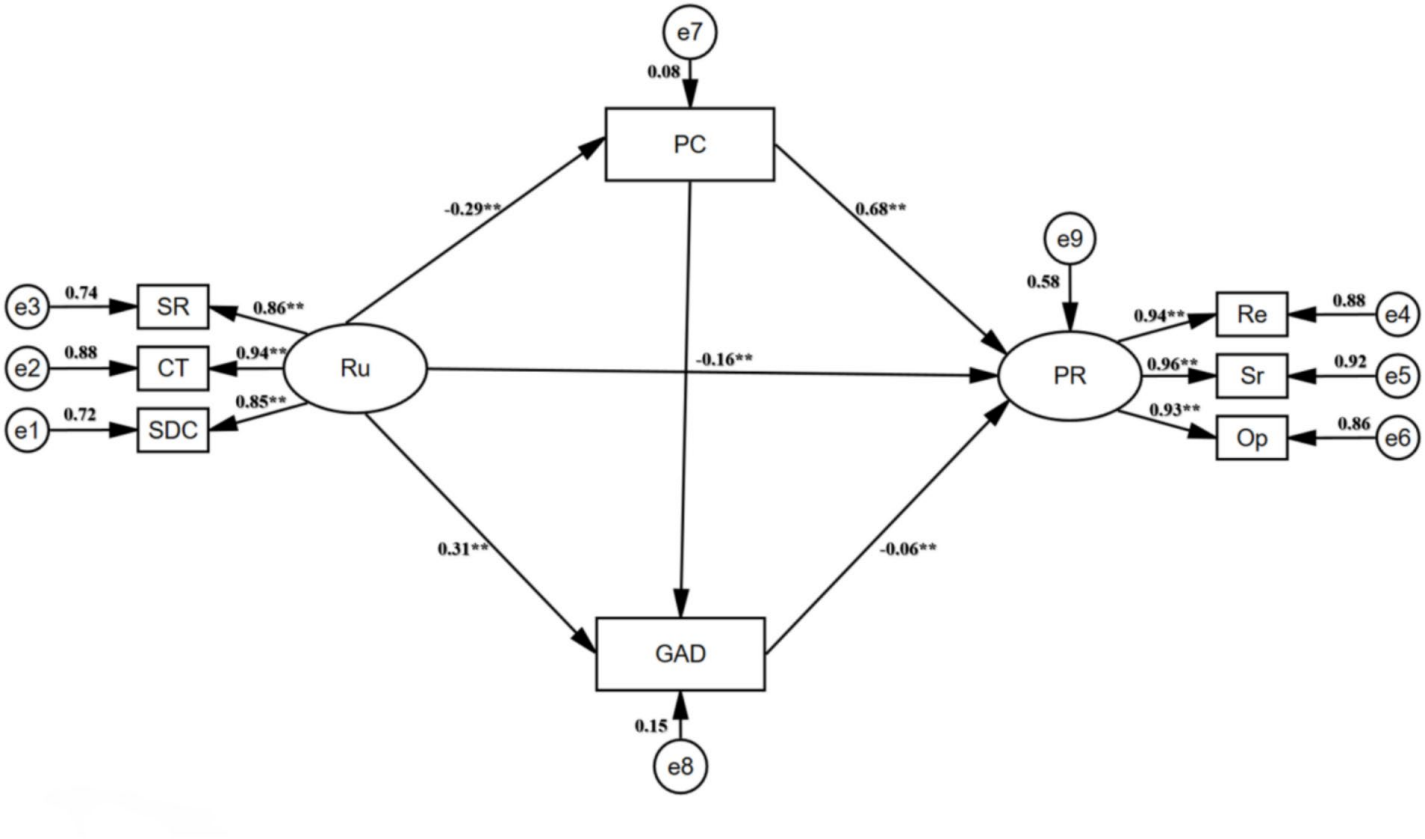
Chain Mediation Model of Rumination on Psychological Resilience. **p < 0.01. Ru, Rumination; SR, Symptom rumination; CT, Compulsive thinking; SDC, Self-reflection and deep contemplation; PR, Psychological resilience; Re, Resilience; Sr, Self-reliance; Op, Optimism; GAD, Generalized Anxiety Disorder; PC, Proactive Coping.

The above results supported the four hypotheses of this study, that is, rumination can not only directly predict the psychological resilience of civil aviation flight students but also indirectly affect psychological resilience through single or chain paths by inhibiting proactive coping and enhancing generalized anxiety. It is worth noting that the confidence intervals of the total mediation effect, direct effect, and each indirect effect did not include 0, confirming the multiple mediation model (Figure 2).

## 4 Discussion

### 4.1 The direct relationship between rumination and psychological resilience in civil aviation flight students

This study first supported its core hypothesis (H1), demonstrating that rumination is a significant negative predictor of psychological resilience among civil aviation flight students. This finding aligns closely with the central tenets of the Conservation of Resources (COR) theory, which posits that individuals strive to acquire, protect, and maintain resources in the face of stress. A continuous net loss of these resources can trigger a “resource loss spiral,” culminating in a decline in adaptive functioning.

Rumination is, in essence, a maladaptive cognitive process that constitutes an inefficient drain on valuable psychological resources (Nolen-Hoeksema et al., 2008). When individuals repeatedly dwell on the causes, processes, and consequences of negative events without taking effective action, this cognitive pattern becomes particularly detrimental, especially within high-stress occupational groups. For example, research among military veterans has shown that rumination is significantly associated with lower levels of resilience and undermines the capacity of resilience to buffer against the development of post-traumatic stress disorder (PTSD) (Blackburn and Owens, 2016). Similarly, a study on clinical nurses found that work-related rumination led directly to diminished psychological flexibility and the adoption of more rigid coping strategies (Üzar-Özçetin and Budak, 2024). Collectively, this body of research indicates that elevated levels of rumination represent a significant risk factor for diminished psychological resilience.

To further contextualize the significance of these findings within aviation, we draw upon the Job Demands-Resources (JD-R) model (Bakker and Demerouti, 2007), which has been widely applied to high-stakes occupations such as Air Traffic Controllers (Alaydi and Ng, 2024). Within the JD-R framework, the environment of flight students is characterized by immense “Job Demands,” including high-intensity training, strict “zero-error” evaluation standards, and a pervasive fear of failure. These demands place continuous strain on students’ cognitive and emotional regulatory capacities. In this context, rumination is not merely a byproduct of negative emotion but a highly destructive, maladaptive strategy that actively depletes “Personal Resources” (Li et al., 2024a; Milaad et al., 2025). This resource depletion manifests specifically as an occupation of executive cognitive functions. Flight operations, particularly in complex or emergency scenarios, rely heavily on finite cognitive resources such as sustained attention, working memory, decision-making, and interference control (Ribas et al., 2010). Cognitive science research confirms that rumination is a cognitively demanding process that continuously consumes attentional and working memory resources (Sp and Chandramohan, 2008).

This performance decline resulting from the depletion of cognitive resources is corroborated in other high-risk, high-reward professions. For instance, research on financial traders has shown that individuals who exhibit strong emotional reactions to gains and losses—a response closely linked to rumination—demonstrate significantly poorer trading performance (Lo et al., 2005). Similarly, among surgeons, who face immense pressure and have zero tolerance for error, maladaptive coping styles have been tied to increased burnout and adverse patient outcomes (Spiliopoulos et al., 2014; Vohra, 2018). This cross-disciplinary evidence reinforces a central point: in any field where outcomes depend on clear, rapid, and accurate decision-making, rumination ceases to be a mere mental health issue and becomes a direct threat to core operational capability.

Therefore, the findings of this study extend beyond psychological observation and have direct relevance for the critical domains of human factors and aviation safety. They reveal that rumination operates as a cognitive-level safety hazard, directly eroding psychological resilience by depleting a flight student’s most valuable asset: the ability to maintain clarity of thought under pressure. This implies that interventions targeting rumination are not simply a matter of mental health support; they are a crucial flight safety measure, on par with technical training and procedural optimization.

### 4.2 The mediating role of proactive coping and generalized anxiety between rumination and psychological resilience in civil aviation flight students

This study revealed that proactive coping is a significant mediating pathway through which rumination affects psychological resilience, thereby supporting Hypothesis H2. Notably, this pathway accounted for the majority of the total indirect effect, highlighting the pivotal role of proactive coping in protecting civil aviation flight students from the detrimental impact of rumination. This aligns with existing research showing that rumination not only directly diminishes psychological flexibility but also weakens adaptive capacity by undermining the use of proactive coping strategies (Li et al., 2024b).

Proactive coping is a future-oriented style aimed at building and accumulating resources to manage challenges and foster personal growth (Xu et al., 2022b). In pilot populations, it has been shown to directly promote mental health and to exert an indirect protective effect by lowering perceived stress (Xu et al., 2022b). It constitutes a crucial “Personal Resource” within the JD-R model and serves as a cornerstone of psychological resilience. The role of proactive coping as a protective factor for resilience is well-supported, with specific strategies like problem-focused coping and positive cognitive reappraisal known to significantly enhance resilience levels (Jin et al., 2016; Zhang, 2015).

Within the “zero-error” aviation safety culture, demands for perfection can reinforce ruminative tendencies through catastrophic thinking. This may lead to a severe depletion of proactive coping resources, creating a vicious cycle of “cognitive resource depletion leading to coping strategy failure.” The unique stressors of flight training, such as the sharp surge in cognitive load required to manage in-flight emergencies, can exacerbate the drain on attentional resources caused by rumination, thereby impairing the effective execution of standard operating procedures (Dismukes et al., 2017). Therefore, interventions designed to enhance resilience in flight students should focus on restructuring cognitive appraisal systems. We recommend the development of Stress Inoculation Training (SIT) that is integrated with Crew Resource Management (CRM) principles, as well as the establishment of peer support programs that encourage and normalize self-disclosure (Truong et al., 2019).

Furthermore, this study found that generalized anxiety has a significant mediating effect on the relationship between rumination and psychological resilience, supporting Hypothesis H3. By sustaining a focus on negative information and potential threats, rumination significantly exacerbates anxiety levels among flight students (Segerstrom et al., 2000), and this elevated anxiety, in turn, erodes their psychological resilience. This finding is consistent with an extensive body of research. Rumination is considered a core maintenance factor for generalized anxiety, as it induces a state of continuous vigilance and worry through an excessive focus on uncertainty and catastrophic interpretations (Harrington and Blankenship, 2002).

The core of psychological resilience is characterized by adaptability in the face of adversity, which includes effective problem-solving and the capacity to recover from stress (Luthar et al., 2000). In contrast, the persistent, pervasive worry that defines generalized anxiety severely disrupts these normal protective processes (Borkovec et al., 2004). It undermines an individual’s problem-solving confidence and self-efficacy, making them more likely to adopt avoidant, rather than active, coping strategies when confronted with new challenges (Rutter, 1985). For civil aviation flight students, high-stakes events such as licensing examinations are significant stressors. When compounded by high anxiety triggered by rumination, students face a dual drain on their psychological resources, severely impairing their adaptive potential and ultimately diminishing their resilience.

Previous research provides a roadmap for intervention. For example, the targeted enhancement of key psychological processes such as mindfulness, metacognitive awareness, and positive emotions can significantly foster resilience (Robertson et al., 2015). The six-strategy resilience training program proposed by Henderson and Milstein (2003) also offers valuable insights for developing interventions. For civil aviation flight students, we recommend supplementing routine group counseling with a diverse range of activities, including individualized counseling, thematic workshops, and experiential training. Intervention plans should incorporate principles from positive psychology, mindfulness training, and stress management to meet the diverse needs of students, thereby enhancing the appeal, engagement, and efficacy of these programs.

### 4.3 The chain mediating effect of proactive coping and generalized anxiety between rumination and psychological resilience in civil aviation flight students

This study revealed a chain mediation mechanism, supporting Hypothesis 4, whereby rumination impairs psychological resilience by first diminishing proactive coping and subsequently exacerbating generalized anxiety. The finding that rumination negatively predicts proactive coping is consistent with Response Styles Theory, which posits that a repetitive focus on negative events consumes cognitive resources and impedes the generation of problem-oriented strategies (Nolen-Hoeksema et al., 2008). In turn, the link between depleted coping and heightened anxiety is also well-supported, as proactive coping—a future-oriented style that promotes action and personal growth—is a known protective factor against anxiety (Anagnostopoulos and Griva, 2012; Tielemans et al., 2015).

Interpreted through the lens of the Self-Regulatory Executive Function (S-REF) model (Matthews and Wells, 2000), our findings suggest that rumination triggers a vicious cycle for flight students. Persistent negative thought inhibits the activation of proactive coping, which blocks access to external resources and, through a corresponding increase in generalized anxiety, creates a pattern of anxiety activation specific to the aviation context. A disruption of this dynamic equilibrium further erodes psychological resilience. Elevated anxiety can lead to dysfunction in emotion regulation processes (Chilver et al., 2020) and greater cognitive rigidity during stressful events (Cavanagh and Frank, 2014), culminating in a self-perpetuating cycle of “rumination → coping resource depletion → anxiety amplification → resilience impairment.” Therefore, this cascading depletion mechanism can be understood within the Conservation of Resources theory, where it manifests with a unique characteristic in the aviation domain: the cumulative effect of micro-losses from high-frequency simulation training (Hobfoll, 1989). This specific contextual factor may explain how this chain mediation pathway, even if composed of individually modest effects, can exert a substantial long-term impact on psychological resilience.

Meanwhile, the core cultural concept of “face” (mianzi) functions as a critical “amplifier” within this study’s model. In Chinese society, “face” is a central social and moral construct linked to an individual’s reputation, prestige, and social status (Prutskikh and Merkulova, 2022). Research indicates that the concept of “face” in Chinese culture significantly influences mental health attitudes and help-seeking behaviors (Wang and Zhang, 2021). Concerns about face, along with the related notion of “fear of losing” (pa shu), also affect feedback-seeking behaviors in educational settings (Hwang et al., 2002). In the high-achievement, “zero-error” context of civil aviation flight training, a public examination failure extends far beyond a mere technical setback. It is more likely to be interpreted as a significant loss of face, an event that not only damages personal reputation and induces shame (Yang and Kleinman, 2008) but also indirectly impacts mental health through experiences of anxiety (Chiu et al., 2015; Mak et al., 2015). This process internalizes an objective, external event into persistent and debilitating psychological distress, ultimately eroding the psychological resilience of civil aviation flight students. Although the concept of face is not exclusive to Chinese culture, it remains a vital factor for understanding psychological distress within the Chinese population (Song, 2018). Consequently, the same psychological mechanisms may manifest with greater intensity and result in more severe impairments within the Chinese cultural context.

To counteract the cycle of psychological attrition amplified by the “face” culture among civil aviation flight students, a multi-level, systematic intervention framework must be constructed. First, at the institutional support level, civil aviation authorities and aviation academies should establish an integrated psychological support system. This system would proactively identify risks through regular psychological assessments and provide adjustment strategies tailored to individual personality traits. The critical objective is to frame mental health support as an integral component of professional competency, rather than a marker of personal weakness. This approach protects the students’ “face” and encourages them to proactively manage their emotional states. Second, at the individual skills intervention level, aviation academies ought to design specialized psychological curricula that directly target rumination as the core mechanism. By introducing evidence-based techniques such as Cognitive Behavioral Therapy (CBT) and mindfulness meditation (Dray et al., 2017; Kral et al., 2018), these programs would equip students with the skills to accurately identify and interrupt the vicious cycle of negative rumination. Concurrently, they would strengthen cognitive control and the capacity for positive thinking, thereby fundamentally enhancing psychological resilience for adapting to high-pressure environments.

## 5 Research implications

This study is the first to validate the psychological mechanism whereby rumination influences psychological resilience through three distinct pathways among civil aviation flight students, providing a crucial foundation for developing a resilience intervention framework tailored to the specific demands of the aviation profession. In terms of clinical practice, our findings offer clear guidance for mental health professionals. The research underscores the urgent need for the early identification of and intervention for rumination among flight students. Furthermore, the validated chain-mediating pathway identifies specific therapeutic targets, suggesting that interventions should not treat anxiety in isolation but rather employ a systemic approach. For instance, Cognitive Behavioral Therapy (CBT) and mindfulness techniques can be utilized to interrupt the ruminative cycle, while problem-solving-oriented training can bolster proactive coping, a core protective factor. This culminates in a comprehensive psychological support program that encompasses prevention, intervention, and ongoing support.

Although this study focuses on Chinese civil aviation flight students, its findings may be generalizable to other occupational groups that face high cognitive loads, stringent performance demands, and significant safety risks, such as military veterans, first responders, surgeons, financial traders, air traffic controllers, and military pilots. These cohorts share similar professional characteristics, necessitating the maintenance of a stable psychological state and optimal cognitive function under high-pressure conditions. However, the profound influence of cultural context on cognitive patterns, coping strategies, and emotional regulation warrants careful consideration. The core concept of “face” (mianzi) in Chinese culture may shape stress expression and the selection of coping strategies among flight students. For example, individuals from Chinese backgrounds may be more prone to experiencing self-criticism and shame or may be less inclined to seek psychological help for fear of being perceived as “weak.” These cultural traits could amplify the negative relationship between rumination and psychological resilience. Compared with findings from Western research, the same psychological mechanisms might manifest with greater intensity and result in more severe impairments within the Chinese cultural context. Therefore, cross-cultural research is essential for validating the universality of our findings and for informing the development of more culturally sensitive psychological intervention strategies.

## 6 Research limitations and future directions

Results of this study must be considered with regards to its limitations. Firstly, our exclusive reliance on self-report measures for data collection introduces several potential sources of bias. In a cohort sensitive to performance and mental health, social desirability bias may have led participants to under-report psychological issues or over-report proactive coping. Consistency and self-perception biases could have also affected data accuracy. Although we tested for common method bias, the potential for such influences cannot be entirely ruled out. Secondly, the study’s cross-sectional design precludes the establishment of causal relationships, allowing only for the identification of associative patterns among variables. Thirdly, the cultural specificity of our sample limits the generalizability of the findings. Conceptions of mental health, coping strategies, and modes of emotional expression within the Chinese cultural context may differ from those in other cultures, thereby affecting the strength and nature of the observed relationships. Fourthly, the study did not adequately account for the influence of group differences, such as variations in career development stages or work tenure.

Based on these limitations, future research can be further expanded and deepened in several directions. In terms of research methodology, we recommend adopting diversified measurement approaches by incorporating objective measures such as physiological indicators, behavioral observations, and third-party assessments to reduce single-method bias. Longitudinal research designs should be employed with multi-wave follow-up surveys to explore the dynamic developmental trajectories and causal relationships among psychological resilience, coping strategies, and mental health. Cross-cultural comparative studies should be conducted to verify the applicability and universality of the research model across different cultural contexts. Regarding research content, future studies could deeply explore other potential mediating and moderating variables, such as work values, perceived organizational support, and work-life balance. Attention should be paid to the differential characteristics of groups at different career development stages, with varying years of work experience, and at different job levels. Qualitative research methods should be integrated to deeply explore individuals’ subjective experiences and meaning construction during the coping process. For practical applications, we recommend conducting intervention pilot projects based on research findings, such as psychological resilience training and coping skills development programs, to validate the practical value of theoretical models and provide empirical foundations for constructing more scientifically effective mental health support systems.

## 7 Conclusion

1. Rumination and psychological resilience showed a significant negative correlation, validating the core concept of the Conservation of Resources theory, that is, rumination forms a resource depletion spiral, weakening the psychological resilience of civil aviation flight students.
2. Proactive coping exhibited a significant mediating effect between rumination and psychological resilience. Rumination hinders the development of psychological resilience in civil aviation flight students by suppressing problem-solving ability and cognitive reappraisal strategies.
3. Generalized anxiety exhibited a significant mediating effect between rumination and psychological resilience. Rumination significantly weakens the development of psychological resilience in civil aviation flight students by continuously focusing on negative information and suppressing emotional regulation ability, exacerbating generalized anxiety.
4. Proactive coping and generalized anxiety showed a significant chain mediating effect between rumination and psychological resilience. Rumination indirectly weakens the psychological resilience of civil aviation flight students through a dual-path mechanism of reducing proactive coping levels and exacerbating generalized anxiety.

## Data Availability

The original contributions presented in the study are included in the article/Supplementary material, further inquiries can be directed to the corresponding author.
